# Carbon Nanotubes Hybrid Hydrogels for Environmental Remediation: Evaluation of Adsorption Efficiency under Electric Field

**DOI:** 10.3390/molecules26227001

**Published:** 2021-11-19

**Authors:** Giuseppe Cirillo, Manuela Curcio, Lorenzo Francesco Madeo, Francesca Iemma, Giovanni De Filpo, Silke Hampel, Fiore Pasquale Nicoletta

**Affiliations:** 1 Department of Pharmacy, Health and Nutritional Sciences, University of Calabria, 87036 Rende, Italy; manuela.curcio@unical.it (M.C.); francesca.iemma@unical.it (F.I.); fiore.nicoletta@unical.it (F.P.N.); 2 Leibniz Institute of Solid State and Material Research Dresden, 01069 Dresden, Germany; l.f.madeo@ifw-dresden.de (L.F.M.); s.hampel@ifw-dresden.de (S.H.); 3 Department of Chemistry and Chemical Technologies, University of Calabria, 87036 Rende, Italy; giovanni.defilpo@unical.it

**Keywords:** wastewater treatment, hybrid hydrogel, carbon nanotubes, isothermal studies, kinetic studies

## Abstract

The performance of Carbon Nanotubes hybrid hydrogels for environmental remediation was investigated using Methylene Blue (MB), Rhodamine B (RD), and Bengal Rose (BR) as model contaminating dyes. An acrylate hydrogel network with incorporated CNT was synthesized by photo-polymerization without any preliminary derivatization of CNT surface. Thermodynamics, isothermal and kinetic studies showed favorable sorption processes with the application of an external 12 V electric field found to be able to influence the amount of adsorbed dyes: stronger interactions with cationic MB molecules ($q_{exp}$ and $q_{exp}^{12}$ of 19.72 and 33.45 mg g^−1^, respectively) and reduced affinity for anionic RD ($q_{exp}$ and $q_{exp}^{12}$ of 28.93 and 13.06 mg g^−1^, respectively) and neutral BR ($q_{exp}$ and $q_{exp}^{12}$ of 36.75 and 15.85 mg g^−1^, respectively) molecules were recorded. The influence of pH variation on dyes adsorption was finally highlighted by reusability studies, with the negligible variation of adsorption capacity after five repeated sorption cycles claiming for the suitability of the proposed systems as effective sorbent for wastewater treatment.

## 1. Introduction

The environmental contamination is becoming the major concern of government regulatory agencies, due to the harmful effects on ecosystems [1,2], with organic dyes used in textile, plastic, wood, paper, and food industries representing the most challenging contaminants for an effective environmental remediation [3,4]. Their chemical structure, often consisting in water soluble aromatic amines, together with the negligible biodegradation profiles, is responsible for severe adverse effects on human health, with kidney, liver, central nervous system, and cancer diseases being the most dangerous disorders [5,6,7].

As a consequence of the increasing demand for effective remediation methods, several different technologies (e.g., ion exchange, electro- and light- degradation, bioremediation, biosorption, and membrane separation) have been established and are continuously upgraded for the removal of toxic components from wastewater [8,9,10].

Biosorption, involving the use of polysaccharide and protein materials from animal or vegetable origin appropriately formulated into different structures [11,12], is a widely explored technology for the removal of organic dyes since it couples the cost-effectiveness and high efficiency of sorption methods with the biocompatibility and sustainability of natural polymers allowing for a reduction environmental impact [13,14,15]. Hydrogels based on natural polymers are emerging as highly effective absorbers due to their ability to store a large amount of water and the possibility to introduce tailored chemical functionalities for the selective interaction with organic contaminants [16,17]. On the other hand, such materials suffer from some severe drawbacks limiting their applicability in industrial practice, mainly related to their poor mechanical properties [18,19]. To overcome these limitations, several attempts have been made for the development of hybrid materials with improved performance consisting in natural polymers crosslinked/inserted into acrylate networks [20,21]. Moreover, great attention has been focused on the development of composite materials where inorganic components such as silica, clay, metal, or carbon nanostructures are incorporated within a polymer network, with the ultimate aim to enhance the adsorption capacity due to the large specific surface area of nanoparticle systems [22,23]. Among others, carbon nanostructures, and carbon nanotubes (CNT) in particular, offer key advantages in the preparation of highly effective sorbent materials because of their hollow and layered surface allowing for high interaction with organic molecules via π–π staking [24,25,26]. On the other hand, the applicability of unmodified CNT in adsorption processes is significantly restricted by the absence of polar functional groups resulting in a poor interaction with hydrophilic compounds [27]. Thus, it is evident the importance of both CNT and polymer counterparts for an effective wastewater remediation, with the modification of CNT surface allowing the enhancement of the eco- and bio- compatibility of the composite sorbents [28,29].

We previously proved the possibility to insert CNT into composite hydrogels by a modified “grafting from” approach consisting in the radical polymerization of acrylamide (AAm), N-N’-ethylenebisacrylamide (EBA) in the presence of Gelatin-coated multi-walled CNT (MWCNT) [30]. The resulting system was evaluated in terms of affinity for charged drugs, and proposed as an electro-responsive delivery device, with the release profiles closely found to be dependent on both the net charge of the drug molecule and the applied external voltage.

The application of an electric field to improve the performance of sorbent materials was extensively explored in the literature, e.g., for the adsorption of asphaltenes at a pendent drop interface [31], or for the adsorption of different types of proteins onto optically transparent carbon electrodes [32]. It was found that the electric-field induced fluid flows help asphaltene molecules to interact with the water−oil interface, and that the adsorption of hard proteins was more susceptible to the application of the external electric field compared to soft proteins, due to the different electric behavior of the two classes of macromolecules.

Within the present study, we aim to test the versatility of such hybrid hydrogels, investigating their ability to adsorb three different organic dyes with different electric behavior such as Methylene Blue (MB), Rhodamine B (RD), and Bengal Rose (BR). For a more exhaustive characterization, the mechanism of dyes adsorption, kinetics, and thermodynamics parameters were determined by suitable empirical models.

## 2. Results and Discussion

### 2.1. Synthesis and Characterization of Hybrid Hydrogels

Different methodologies were proposed for the fabrication of hybrid hydrogels (HG_NT_) based on the incorporation of MWCNT into the polymer networks [33,34,35]. Here, we used a previously reported strategy based on a modified “grafting from” approach involving the co-polymerization of Gelatin (Gel) coated MWCNT (Gel@MWCNT) in the presence of AAm and EBA as plasticizing and crosslinking monomers, respectively.

In detail, individual MWNT of about 20–30 graphene walls (10–30 μm length, 10–80 nm outer diameter, 5–25 nm inner diameter) were firstly synthesized by an aerosol assisted chemical vapor deposition method where cyclohexane and ferrocene acted as a carbon source and metal–organic catalyst precursor, respectively [36]. Then, MWCNT underwent non-covalent functionalization with Gel to improve their stability in water. The strong hydrophobic behavior of pristine materials resulted, indeed, in the formation of big aggregates in water media of 1.70 μm average hydrodynamic diameter (polydispersity index PDI of 0.30). On the other hand, Gel@MWCNT showed a reduced tendency to agglomerate (0.34 µm hydrodynamic diameter, PDI of 0.05) and were thus used for the synthesis of hybrid hydrogels. Gel@MWCNT were inserted in the pre-polymerization feed together with the selected amount of acrylate monomers and photo-polymerized to obtain the hybrid hydrogels HG_NT_, while control samples (HG_B_) were prepared when reaction was conducted using Gel instead of Gel@MWCNT.

As extensively investigated in the literature, the employed synthetic procedure is based on the ability of growing polymer radicals to be linked on the defective site of MWCNT surface, resulting in chain termination and MWCNT immobilization into the polymer network [30]. Moreover, this approach is effective for the incorporation of MWCNT into the hydrogel structure, as confirmed by Scanning Electron Microscopy (SEM), FT-IR, Raman, and Differential Scanning Calorimetry (DSC).

**Figure 1:**
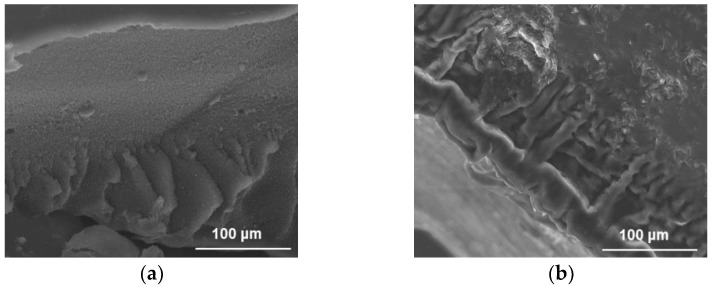
SEM images of (**a**) HG_B_ and (**b**) HG_NT_ samples, showing the presence of MWCNT homogeneously embedded in the polymer network of HG_NT_.

The evaluation of the hydrogel morphology (Figure 1a,b) showed MWCNT well embedded into the HG_NT_ polymer matrix (Figure 1b).

FT-IR (Supplementary Materials Figure S1) and Raman analysis [30] were used to assess the covalent incorporation of MWCNT inside the polymer network. In detail, the FT-IR spectrum of HG_NT_ (Figure S1 in Supplementary Materials) showed the typical CNT band at 1615 cm^−1^ (C=C stretching) [37] in the HG_NT_ spectrum, while the modification in the intensity of D and G bands (I_D_/I_G_ ratio) in Raman spectra of pristine and polymerized MWCNT [30] clearly showed the covalent attachment of polymer chains onto the MWCNT surface. It is well known, indeed, that the D (1304 cm^−1^) and G (1589 cm^−1^) bands are related to the disorder and graphitic characters of MWCNT, respectively. The presence of defects sites on pristine MWCNT surface induced by the purification procedure resulted in an I_D_/I_G_ ratio of around 1.20 [38], becoming 1.80 in the case of HG_NT_, as a confirmation of the increased disorder due to the covalent bonds formation.

Moreover, the covalent incorporation of MWCNT significantly modified the thermal and electrical properties of HG_B_. The endothermic peak (T_m_) found in the HG_B_ DSC thermogram was missed in HG_NT,_ confirming the enhanced thermal stability of the polymer blocks within hybrid hydrogels [39], while the electrical resistance was significantly decreased from values higher than 10^12^ Ω cm^−1^ (HG_B_) to 7.0 10^10^ Ω cm^−1^ (HG_NT_) [30].

### 2.2. Sorption Properties of Hybrid Hydrogels

In the last decades, the huge demand for minimizing the wastewater discharges and mitigating the hazards of pollutants, indeed, carried on to the development of various techniques, including physical, chemical, and biological methods [40]. Each method shows advantages or disadvantages depending on different factors such as dye chemical features, dye concentration, presence of additional impurities, cost of the process [41]. Here, we aim to explore the key advantage of adsorption methods, defined by the United States Environmental Protection Agency as one of the most excellent and best wastewater treatment techniques [42], by coupling the surface properties of CNT, the water affinity and eco-compatibility of acrylate hydrogels with the possibility to affect the sorbent properties by applying an external electric field. Moreover, such materials can open new opportunities for a multi-technique approach: when formulated into hydrogel films, they can be also used in membrane filtration process, another efficient and economically effective approach for wastewater remediation [43], while the intrinsic electrical and photothermal properties of CNT, together with the possibility to decorate their surface with suitable catalysts, can open the possibility to use the hybrid hydrogel as a substrate for chemical methods [44].

In our previous work, the ability of HG_NT_ hybrid materials to modulate the release of ionic drugs upon application of an external electric field was evaluated and ascribed to the peculiar physic-chemical features of either MWCNT and Gel. Here, based on the scientific evidence reporting the applicability of hybrid materials with similar properties to environmental remediation [45,46], we aim to investigate a further extension of the applicability of such materials, exploiting their high affinity for dye adsorption from water solutions.

**Figure 2:**
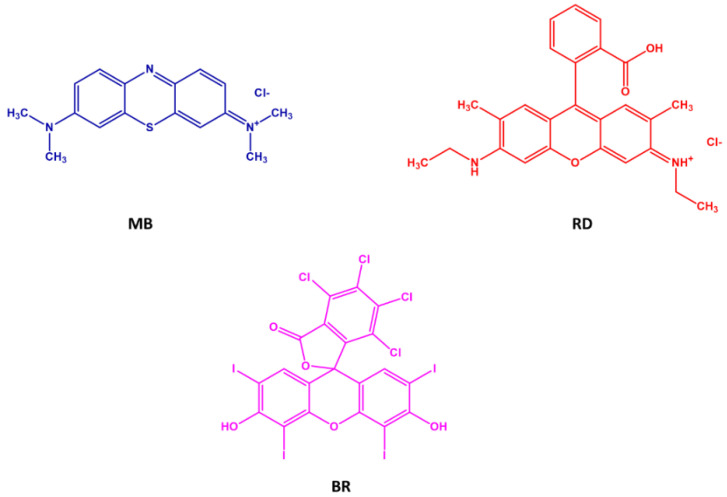
Chemical structure of Methylene Blue (**MB**), Rhodamine B (**RD**), and Bengal Rose (**BR**) dyes.

We extensively investigated the adsorption behavior using three different dyes, namely Methylene Blue (MB), Rhodamine B (RD), and Bengal Rose (BR), either in the presence or in the absence on an applied voltage (Figure 2).

HG_NT_ swelling behavior was investigated in pure water media, obtaining water content percentage (WR) of 267 ± 4%, lower than that recorded for the blank (HG_B_) hydrogel samples (317 ± 3%) because the presence of MWCNT was reported to reduce the hydrophilic behavior due to the hydrophobic π-π CNT surface and enhance the mechanical strength hindering the swelling process [39]. Moreover, the determination of hydrogel porosity (33.24 and 24.67 nm for blank and hybrid hydrogels, respectively) by the empirical model consisting in the Kulkarni’s version of Flory–Rehner theory [47], allowed hypothesizing of the ability of hybrid materials to adsorb organic molecules within the polymer network.

**Figure 3:**
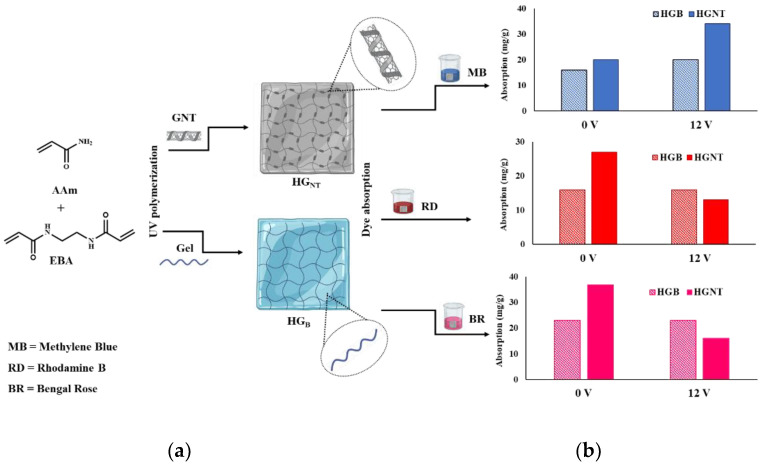
(**a**) Schematic representation of synthesis of hybrid hydrogels and (**b**) dye adsorption performance at different voltage conditions.

The schematic representation of hydrogel synthesis and sorption processes were depicted in Figure 3.

At 0 V conditions, it is clearly evident that the presence of CNT induces higher affinity of HG_NT_ for any tested dyes, with the amount of adsorbed dye ($q_{exp}$) being 19 mg g^−^^1^ (MB), 29 mg g^−^^1^ (RD), and 37 mg g^−^^1^ (BR) becoming 16, 15 and 23 mg g^−^^1^ in the case of HG_B_ samples. These results are in agreement with the literature data claiming for higher loading efficiency of hybrid materials based on carbon nanostructures [48,49].

To calculate the thermodynamic behavior of dye adsorption process, we used an approach reported in the literature where the Gibbs free energy (ΔG°) was described as a function of the $\frac{q_{e}}{C_{e}}$ (L g^−^^1^) ratio [50]. The model allowed calculating ΔG°, the enthalpy (ΔH°), entropy (ΔS°), and according to Equations (1) and (2):

(1) $$∆G^{0}=-RTln\left(K_{D}\right)$$

(2) $$ln\left(K_{D}\right)=\frac{-∆H^{0}}{RT}+\frac{∆S^{0}}{R}$$

Here, *R* is the universal constant of gases (8.314 J mol^−^^1^ K^−^^1^), *T* the absolute temperature (K), and *K_D_* the equilibrium constant calculated as follows (Equation (3)):

(3) $$K_{D}=1000\times \frac{q_{e}}{C_{e}}$$

**Figure 4:**
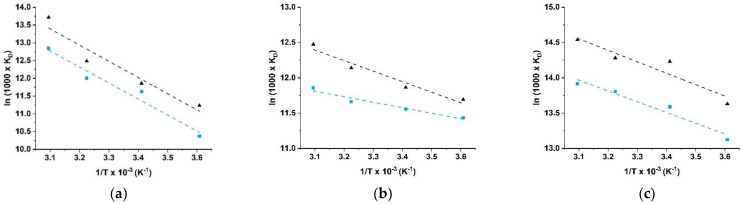
Relation of $\ln\left(1000\times K_{D}\right)$ vs. 1/T to determine thermodynamic parameters for (**a**) MB, (**b**) RD, and (**c**) BR adsorption by HG_B_ (blue lines) and HG_NT_ (black lines).

The linear fits for the three dyes are depicted in Figure 4.

The evaluation of the obtained parameters (Table 1) showed that the adsorption processes were possible, spontaneous, and exothermic (negative ΔG^0^ and ΔH^0^ values) in all cases. The positive ΔS^0^ values indicated that the random collisions of dye molecules increase during the adsorption processes [50].

More in detail, in the tested temperature range, the lowest ΔG^0^ were recorded for BR, while the more exothermic process was found to be the MB adsorption, suggesting a different electronic behavior of the dyes.

Then, the effect of the application of an external electric field (6 and 12 V) on the sorbent performance was explored, with the analysis of the variations (%) of the $q_{exp}$ values at 0 V ($q_{exp}^{0}$) and applied voltage ($q_{exp}^{V}$) being expressed according to the following Equation (4):

(4) $$∆Q^{V}=\left|\frac{q_{exp}^{V}-q_{exp}^{0}}{q_{exp}}\right|\times 100$$

From data in Table 2, it is clear that no significant variations were recorded when HG_B_ was used, while a modification in either nature or intensity of the sorbent/sorbate interactions can be hypothesized upon application of the electric field.

In detail, an enhancement in $q_{exp}^{V}$ occurred in the case of MB, whereas lower amount of RD and BR were retained. The analysis of data in Table 2 suggested that the electric field promotes the formation of negative charge on the hybrid hydrogels (due to the presence of both MWCNT and Gel), thus resulting in stronger interactions with cationic molecules (higher $q_{exp}^{V}$ for MB dye) and reduced affinity for anionic molecules (lower RD adsorption). As far as the neutral BR dye is concerned, the reduced affinity is related to the induction of repulsive forces between polarized sorbent and sorbate molecules, with the latter also undergoing lactone ring opening and formation of negatively charged species [30,39]. These effects were more evident at 12 vs. 6 V, while higher voltages (e.g., 24 V) cannot be tested due to significant (>15%) dyes degradation under these conditions.

When comparing the effectiveness of HG_NT_ hybrid hydrogels in absorbing the dyes with the carbon nanomaterials-based systems available in the literature, it found that our system possessed similar adsorption capacity for BR (5.98 mg g^−^^1^ [51] and 6.22 mg g^−^^1^ [52]), while considerable low affinity for MB (from 35.4 to 188.68 mg g^−^^1^ [53]) and RD dyes (from 55.56 to 963.04 mg g^−^^1^ [54,55]). This can be ascribed to the peculiar characteristics of our systems where only 0.5% (by weight) of CNT are incorporated and their surface, being coated with Gel, is not fully available for dyes adsorption. Nevertheless, the feasibility of the synthetic strategy offers possibility to modify the hydrogel composition to enhance the sorbent performance, with the ability to modify the affinity for the different dyes upon application of the external voltage can open great opportunity for the development of effective sorbent materials.

### 2.3. Isotherm Data Analysis

For a proper understanding of an adsorption process (e.g., physical or chemical phenomena, mono- or multi- layered processes, homogeneous or heterogeneous sorption), and the design of effective systems with optimized parameters, the evaluation of relationships between adsorbent and adsorbate at equilibrium is of paramount importance [56,57]. Here, we investigated such relationship in the absence of electric stimulation and in the presence of the higher tested voltage (12 V) for either HG_B_ or HG_NT_ samples.

**Figure 5:**
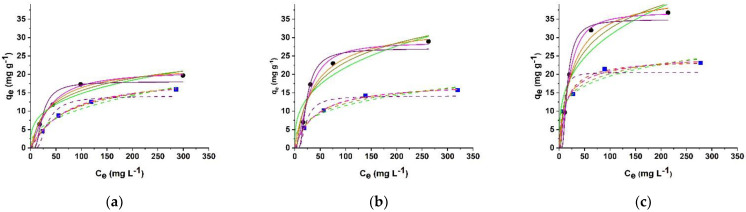

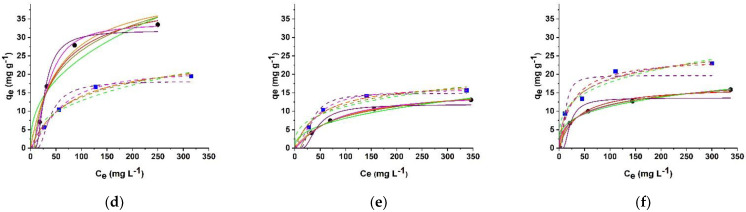
Langmuir (Red lines), Freundlich (Green lines), Red–Pet (Orange lines), Sips (Magenta lines), Dub–Rad (Purple lines), and Temkin (Dark Yellow lines) adsorption isotherms for (**a**,**d**) MB, (**b**,**e**) RD, and (**c**,**f**) BR dyes by HGNT (solid lines) and HGB (dashed lines) under (**a**–**c**) 0 and (**d**–**f**) 12 V conditions. (circles) and (squares) are the experimental data for HG_NT_ and HG_B_, respectively.

As expected, in all cases, an increased initial dye concentration was found to lead to increased adsorption capacity by virtue of the enhancement of the concentration gradient at the sorbent/sorbate interface (Figure 5) [58].

Among the different models proposed in the literature to describe such behavior, Langmuir, Freundlich, Redlich–Peterson, Sips, Dubinin–Radushkevich (Dub–Rad), and Temkin models (Equations (S1)–(S6) in Supplementary Materials) were used [59,60]. To avoid any distortions created in the original error distribution, Origin software was used for the application of the nonlinear optimization method instead of linear regressions [60].

The obtained parameters were collected in Table 3.

In the Langmuir model (Equation (S1)), firstly designed for describing gas–solid phase adsorptions, the dynamic adsorption and desorption equilibrium is described as the balance between the extent of available (adsorption favoured) and covered (desorption favoured) sorbent surface [61].

The model assumes the adsorption as an ideal monolayer process, with a fixed number of binding sites available on the surface of the adsorbent and no interaction between adsorbed species. As a consequence of this statement, when occupied by a sorbate molecule, no further adsorption can take place in a specific sorbent site [62]. In this model, the equilibrium parameter *R_L_* (Equation (S7) in Supplementary Materials) is the essential feature denoting the adsorption nature to be unfavourable (*R_L_* > 1), linear (*R_L_* = 1), favourable (0 < *R_L_* < 1), or irreversible (*R_L_* = 0). On the other hand, heterogeneous sorbent systems are described by Freundlich model (Supplementary Materials Equation (S2)), with the heterogeneity factor *n_F_* indicating favourable (0 < 1/*n_F_* < 1), irreversible (1/*n_F_* = 1) or unfavourable (1/*n_F_* > 1) processes [62].

Better mathematical fits were recorded for Langmuir model as per higher R^2^ values in Table 3, clearly suggesting a predominant surface adsorption mechanism, with heterogenous contribution of Freundlich model being evident in the case of BR adsorption by HG_NT_ under 12 V conditions (R^2^ of 0.9867).

Favourable sorption processes are evoked by both models (0 < *R_L_* < 1 and 0 < 1/*n_F_* < 1), proving the suitability of the hybrid hydrogels as effective sorbent materials. Moreover, the variation of *q_max_* values in the Langmuir model upon application of the external voltage clearly showed the electro-responsivity of the hybrid hydrogels: an increase *q_max_* is recorded when MB is used as dye, while an opposite trend occurred for RD and BR. As expected, the variation of *q_max_* by the electric field is the result of the incorporation of MWCNT, since the extent of *q_max_* variation is significantly higher in HG_NT_ than in HG_B_ cases. The Freundlich model is consistent with this statement, since the *k_F_* parameter values showed a similar behavior.

Since a monolayer sorption process is assumed, we can use the maximum dye adsorption to estimate the Apparent Specific Surface Area (SSA) of HG_B_ and HG_NT_ according to the Equation (S8) in the Supplementary Materials [63]. Literature data suggested that the areas covered per dye molecule are 1.38 10^−18^ m^2^ for MB [63], 3.48 10^−19^ m^2^ for RD [64], and 5.0 10^−19^ m^2^ for BR [65], resulting in SSA of 13059 (MB), 3329 (RD), and 6953 (BR) m^2^ g^−1^ in the case of HG_B_, and 16052 (MB), 5778 (RD), and 11061 (BR) m^2^ g^−1^ when HG_NT_ is used as sorbent. As expected, different values were obtained for the three dyes, since this evaluation is based on the maximum adsorption capacity, and thus is strongly affected by the different dyes to sorbent interactions. In our conditions, since MB covers the wide hydrogel surface, we can use the value obtained for this dye to estimate the SSA of hydrogels. Moreover, this estimation can be used as a direct evidence of the different sorbate to sorbent affinity, and helps in understanding the effect of applying the external voltage. Low variations were indeed recorded for HG_B_ samples (SSA of 15932 (MB), 3302 (RD), and 6919 (BR) m^2^ g^−1^), while a significant enhancement in the HG_NT_ SSA was obtained for MB sorption process (27339 m^2^ g^−1^) as a result of both the higher swelling degree of the hydrogel matrix (341%) and the enhanced sorbate to sorbent affinity. A totally different behavior was recorded for the RD and BR sorption processes, where the decrease of the apparent SSA to 2780 (RD) and 4816 m^2^ g^−1^ (BR) was assigned to the insurgence of the repulsion forces between hybrid hydrogels and dyes, and not yet to the changes in hydrogels water affinity.

More information can be obtained by the application of Red–Pet model (Supplementary Materials Equation (S3)), often used as a compromise between Freundlich and Langmuir systems in accordance to the *g* value lying between 0 and 1: a Langmuir isotherm is indeed obtained when *g* = 1, while $\alpha_{RP}C_{e}^{g}\gg 1$ accounts for the Freundlich model [66]. Data in Figure 5 (orange lines) and Table 3 proved the suitability of this model in describing the adsorption of all dyes, with R^2^ > 0.94 in all experimental conditions. From the analysis of the sorption capacity *q_RP_* (calculated by Equation (S9) in Supplementary Materials) is evident the higher affinity of hybrid hydrogels for all the tested dyes at 0 V conditions (higher *q_RP_* for HG_NT_ vs. HG_B_) [60]. Moreover, *q_RP_* helps in understanding the effect of the external voltage on the sorption efficiency of the samples. By comparing the value obtained for HG_NT_ at 12 vs. 0 V conditions, a significant enhancement of sorption capacity was recorded for MB, while the opposite trend was shown for RD and BR dyes, suggesting that the electronic perturbation of the MWCNT surface induced by the electric field increased the affinity for MB while hindered the interactions with the other two dyes. As expected, the variations recorded for HG_B_ samples were less significant.

The Sips isotherm (Figure 5, Magenta lines) is another three-parameter model applied for describing the sorption process (Supplementary Materials Equation (S4)) as a combination of Langmuir and Freundlich ones [60]. Here, *n_s_* = 1 and *C_e_* (or *k_s_*) close to 0 result in Langmuir and Freundlich equations, respectively. The calculated parameters (Table 3) showed that the sorption process is intermediate between the two limit models, approaching to Langmuir, especially for MB and RD dyes, while in the case of BR, a more relevant contribution of Freundlich model was recorded as per lower *n_s_* values. This is in agreement with the data obtained from Supplementary Materials Equation (S2) (Table 3), showing R^2^ > 0.90 in the case of BR sorption from both HG_B_ and HG_NT_.

Dub–Rad (Supplementary Materials Equation (S5)) is a more general model, not assuming homogeneous or heterogeneous surfaces, and useful to understand the nature (chemical vs. physical) of the adsorption process. In this model, the key determining parameter is the apparent energy of adsorption mechanism (E, Equation (S11) in Supplementary Materials), indicating chemical (8 kJ mol^−1^ < E < 16 kJ mol^−1^) or physical (E < 8 kJ mol^−1^) process [60]. The fitting of the experimental data and the related parameters was depicted in Figure 5 (Purple lines) and Table 3. The model was found to be suitable for describing the adsorption of the three dyes by both blank and hybrid hydrogels (R^2^ > 0.87, see Table 3), with the obtained E values claiming for a predominant physical phenomenon in all cases.

Finally, the Temkin model (Supplementary Materials Equation (S6)) was applied to the experimental data (Figure 5, Dark Yellow lines). As seen for Dub–Rad isotherm, this model focus on the free energy of the sorption process (B), which is assumed to be a function of surface coverage and can be expressed according to the Equation (S12) in Supplementary Materials. Temkin parameters (Table 3) were consistent with both Dub–Rad and thermodynamic models, since it is reported that positive B values indicated a physical and exothermic sorption process [60].

### 2.4. Kinetic Data Analysis

Adsorption rate at 0 and 12 V conditions was also investigated in detail, showing that the concentration gradient at the sorbent/sorbate interface progressively hinders more of dye molecules to enter the adsorbent with time, thus resulting in fast adsorption at the first experimental times, followed by a slower rate eventually attaining equilibrium. At the first experimental time, a large number of empty sites were available for dyes adsorption, while the repulsive forces between the dye molecules on sorbent surface makes the remaining sites difficult to be occupied [45].

The adsorption rate was investigated by six models describing pseudo-first order, pseudo second-order, Avrami, fractional power, intraparticle diffusion, and Elovich kinetics (Equations (S13)–(S18) in Supplementary Materials). To evaluate the fitting between experimental data, we used the R^2^ and the $\chi^{2}$ values, the latter being calculated according to Equation (S19) in Supplementary Materials.

**Figure 6:**
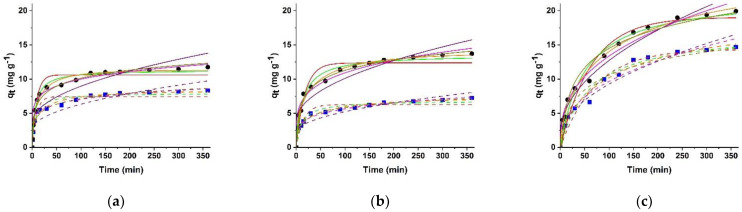

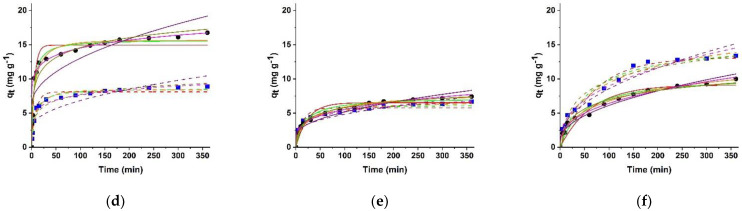
Pseudo-first order (Red lines), Pseudo-second order (Green lines), Avrami (Orange lines), Fractional Power (Magenta lines), Intraparticle Diffusion (Purple lines), and Elovich (Dark Yellow lines) kinetics models for (**a**,**d**) MB, (**b**,**e**) RD, and (**c**,**f**) BR dyes by HG_NT_ (solid lines) and HG_B_ (dashed lines) under (**a**–**c**) 0 and (**d**–**f**) 12 V conditions. (circles) and (squares) are the experimental data for HG_NT_ and HG_B_, respectively.

Primarily, pseudo-first order (Supplementary Materials Equation (S13)) [67] and pseudo-second order (Supplementary Materials Equation (S14)) models [68] were considered (Figure 6, Red and Green lines). Supplementary Materials Equation (S13) describes the adsorption rate based on the adsorption capacity, while Supplementary Materials Equation (S14) claims for sorption process with the involvement of electron sharing and/or exchange between the sorbent and the sorbate [69].

In our experimental conditions, a better fitting was recorded when Equation (S14) was applied to MB and RD adsorption, as per both higher R^2^ and lower $\chi^{2}$ values (Table 4).

The comparison of the results of pseudo-first and pseudo-second order models suggested that, although the sorption process is predominantly physical, the electronic behavior of both hydrogel surface and dye molecules plays a crucial role in determining the sorbent/sorbate interactions. This can be attributed to the properties of carbon nanostructures and is consistent with literature data showing that pseudo-second order kinetics well described the adsorption of RD and MB dyes on graphene nanosheets and CNT, respectively [45,70]. A different behavior was obtained in the case of BR, where, although pseudo-second model kinetics possessed the higher R^2^ values, the better fitting between *q_exp_* and *q_e_* (lower $\chi^{2}$ values) was obtained for the pseudo-first order model, suggesting a lower involvement of electronic perturbation due to the absence of electrostatic charge on the dye molecule.

The whole of the above reported data suggested that the sorption mechanism is a complex phenomenon involving multiple kinetic orders that can change during sorbent/sorbate interactions [71]. Thus, we applied the Avrami kinetic model (Supplementary Materials Equation (S15)) to the experimental data, since the Avrami exponent *n* is a clear indicator of the changes in the adsorption mechanism during the entire process [72]. As a result, a better fitting between experimental and theoretical data was reached in the case of MB and RD, while for BR, higher $\chi^{2}$ values suggested less variable sorption kinetics (Table 4). The fractional power model (Supplementary Materials Equation (S16)) agrees with this statement, as per similar trend in the recorded $\chi^{2}$ values, thus claiming for a similar nature of MB/RD to hydrogels interactions, while different kinetics are involved in the case of BR (Table 4).

The application of intraparticle diffusion model (Supplementary Materials Equation (S17)) aims to elucidate the contribution of the diffusion of the dye molecules within the hydrogel network [58]. It was found that the sorption is mainly a surface phenomenon for the charged MB and RD dyes, while the diffusion is more involved in the BR sorption process (see R^2^ values in Table 4), although the higher $\chi^{2}$ values suggested that the phenomenon is mainly confined to the sorbent surface also in this case.

Finally, information about the correlation between the initial adsorption rate constant and rate constant at any stage of the process are given by the Elovich model (Supplementary Materials Equation (S18)), which can be considered an application of the Temkin isotherm to the analysis of the sorption rate [58]. The results (Table 4), clearly prove that the higher affinity of HG_NT_ samples for all dyes at 0V conditions (higher α values), as well as the electro-responsivity of the hybrid hydrogels. Upon application of the external voltage, indeed, a remarkable increase and decrease in α values are recorded for MB and RD/BR sorption processes, respectively.

### 2.5. Desorption Studies

**Figure 7:**
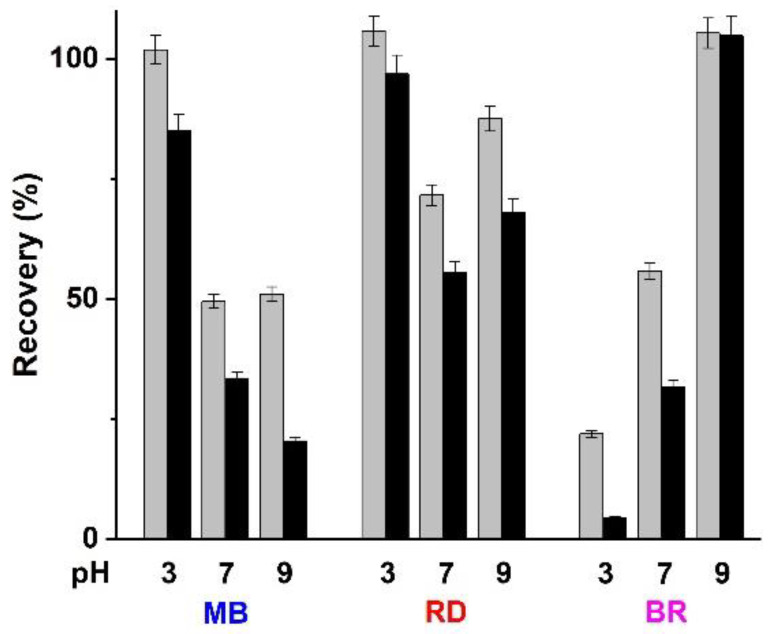
Dyes recovery (%) after 24 h treatment of loaded HG_B_ (grey bar) and HG_NT_ (black bar) with water solutions at different pH values.

The reusability of an adsorbing matrix without significant loss of efficiency is a key important item when considering the potential industrial applications. Consequently, the operational stability of HG_NT_ and HG_B_ samples was evaluated in a repeated batch process. Considering the chemical structure of dyes molecules (Figure 1) and the composition of sorbent hydrogels, after each cycle, hydrogel films were treated with buffered solution at different pH (from 3 to 9) to remove the adsorbed dyes (Figure 7).

It is clearly evident that the variation of pH greatly affected the dye to hydrogels interactions, and that the recovery values are higher in HG_B_ than HG_NT_ case, due to the stronger affinity of MWCNT for all the tested dyes. At acidic pH, a marked decrease in the retention capacity was observed for MB and RD dyes, since the protonation of the COOH functionalities of hydrogel surface reduced the electrostatic interactions with the cationic portions of the two dyes. This effect is even more evident in RD case possessing COOH groups as well. For MB, recovery values below 50% were recorded at neutral and alkaline pH values, as a consequence of the presence of strong dye to hydrogel electrostatic interactions in these conditions.

A different behavior was observed when the BR recovery profiles were investigated. Here, the highest recovery was obtained at pH 9, while a very low amount of dye was detected in the acidic washing media. This can be related to the disruption of hydrogen bonding under alkaline conditions, as well as to the insurgence of electrostatic repulsions between the COO^−^ groups on hydrogel surface and the opened lactone ring of the dye molecule.

Furthermore, since the application of the external voltage was found to significantly decrease the dye to hydrogel affinity in the case of RD and BR, we explored the possibility to use the electric field as desorption agent for these dyes. A fast and complete dyes removal was observed, with the removal (%) reaching values of 97 ± 2% in 1 and 1.5 h for RD and BR, respectively. These data were in agreement with either the isothermal and kinetic adsorption studies of our previous work dealing with the release of therapeutic agents from the same hydrogel system [30]. In detail, a higher affinity of the hydrogel matrix for BR (higher $q_{exp}^{0}$ and kinetic constants) resulted in slower desorption under 12 V condition. Moreover, the same hydrogel was found to totally release the anionic diclofenac sodium after 50 min, thus suggesting that a similar phenomenon occurred in the case of RD and BR. Interestingly, the adsorption capability was found to remain almost unchanged (*p* > 0.05) after five repeated sorption cycles, proving the suitability of the proposed systems as effective adsorbing element for industrial application in wastewater treatment. The effectiveness of HG_NT_ as sorbent materials was also highlighted by comparing the key physic-chemical parameters, e.g., surface appearance and morphology (Figures S2 and S3 in the Supplementary Materials), FT-IR (Figure S1 in the Supplementary Materials) and Raman (Figure S4 in the Supplementary Materials) patterns, as well as electric and thermal properties) before and after the sorption cycles. It was found that when all adsorbed dyes were removed in the proper pH or electric conditions, such parameters (no new peaks in the FT-IR patterns, I_D_/I_G_ value of 1.77, electrical resistance of 7.2 10^10^ Ω cm^−1^, absence of any endothermic peak in the DSC analyses) were unchanged as expected for highly crosslinked acrylic polymer networks.

## 3. Materials and Methods

### 3.1. Synthesis of MWCNT and HG_NT_

MWCNT were synthesized by aerosol assisted chemical vapour deposition method according to the literature [36] The purification procedure involved the thermal treatment of as-grown material at 450 °C in air for 1 h with hydrochloric acid.

For the synthesis of HG_NT_, MWCNT (1.0 mg) were firstly dispersed in a Gelatin (Gel) solution (15 mg, 2.0 mL) by a cup-horn high intensity SONOPULS ultrasonic homogenizer (BANDELIN electronic, Berlin, Germany) with a cylindrical tip operating at 70% amplitude. Then, after the addition of AAm (85 mg) and EBA (100 mg), the solution was purged with N_2_ for 20 min, placed between two 5.0 × 5.0 cm^2^ glass plates, separated with a Teflon spacer (0.6 mm) and brought together by binder clips, and photopolymerized in the presence of Irgacure 2959 as photoinitiator under a Philips HPK 125 high pressure mercury lamp operating at 500 mW cm^−^^2^ and 275 nm wavelength (Philips, Amsterdam, The Netherlands). HG_NT_ samples were washed with distilled water to remove unreacted species and finally dried under vacuum at 40 °C for 12 h [30].

Blank hydrogels HG_B_ were synthesized as reported without adding MWCNT in the pre-polymerization mixture.

All chemicals were from Merck/Sigma Aldrich, Darmstadt, Germany.

### 3.2. Characterization Procedure

The scanning electron microscopy analyses were run on a NOVA NanoSEM 200 (0–30 kV) (Thermo Fisher Scientific, Hillsboro, OR, USA). Samples were cut into thin slices by ultra-microtome cutting technique and deposited onto self-adhesive, conducting carbon tapes (Plano GmbH, Wetzlar, Germany).

The stability of the MWCNT dispersion into the polymerization feed was determined by dynamic light scattering analysis using a 90 Plus Particle Size Analyzer (Brookhaven Instruments Corp, Holtsville, NY, USA) at 25.0 ± 0.1 °C by measuring the autocorrelation function at 90°. The laser was operating at 658 nm.

The Raman spectra were recorded on a Raman Fourier Transform spectrometer IFS 100 (Bruker, Berlin, Germany) preparing samples on an aluminium foil and operating at 633 nm wavelength with a 8 mW laser power.

DSC analyses (25 to 300 °C temperature range, 20 mL min^−1^ nitrogen flow rate, 0.5 °C min^−1^ heating rate) were performed using a DSC200 PC (NETZSCH-Gerätebau GmbH, Selb, Germany) placing samples in aluminium pans hermetically sealed with aluminium lids.

Four points method in the presence of argon pressure at room temperature was used to measure the electric conductivity of powder samples pressed in an insulating ceramic under a constant pressure of 100 MPa.

The swelling degree of hydrogels was investigated in water in the presence and absence of an external electric voltage as follows: specimens of ~1.0 cm^2^ were cut from each sample and placed in a 5-mL sintered glass filter (porosity G3), weighted, and left to swell by immersing the filter in a beaker containing the swelling medium at 37 °C. At suitable time intervals, excess water was removed and samples weighed, after being blotted with a tissue to remove surface moisture.

The water content percentage, WR, was expressed by the following Equation (5):

(5) $$WR=\frac{W_{s}-W_{d}}{W_{d}}\times 100$$

where *W_s_* and *W_d_* are the weights of swollen and dried hydrogels, respectively.

The mean diameters of hydrogels pore (*ξ*) were determined by applying the empirical model proposed in the literature [47] according to the Equations (S20)–(S24) in Supplementary Materials).

### 3.3. Batch Kinetics and Equilibrium Adsorption Studies

The conditions used for dyes adsorption studies were as follows:

(i) isothermal studies: 4.0 mg mL^−1^ HG_NT_, 0.05–0.4 mg mL^−1^ dye solution, contact time 1440 min, temperature 20 °C
(ii) kinetics studies: 4.0 mg mL^−1^ HG_NT_, 0.1 mg mL^−1^ dye solution, contact time 15–1440 min, temperature 20 °C
(iii) thermodynamics studies: 4.0 mg mL^−1^ HG_NT_, 0.1 mg mL^−1^ dye solution, contact time 1440 min, temperature range 4–50 °C

After the reaction time, dyes concentration was determined by UV-Vis analyses on an Evolution 201 spectrophotometer (ThermoFisher Scientific, Hillsboro, OR, USA) operating with 1.0 cm quartz cells, by using the calibration curves of the dye (0.001–0.01 mg mL^−1^).

The experimental amount of adsorbed dye (*q_exp_*, mg g^−1^) was expressed as follows (Equation (6)):

(6) $$q_{exp}=\frac{C_{0}-C_{f}}{m}\times V$$

where *C_0_* and *C_f_* are the initial and final dyes concentration, respectively, *m* is the amount of hydrogels, and *V* is the volume of dye solution.

The adsorption capacity at time t (*q_t_*) and at equilibrium (*q_e_*) were expressed using the Equations (S25) and (S26) in Supplementary Materials.

### 3.4. Hydrogel Regeneration

The regeneration of hydrogel matrices was performed by immersing aliquots (20 mg) of dye loaded hydrogels (as reported in point (i) in Section 3.3) in 100 mL fresh water solutions at different pH conditions, and (ii) as follows: phosphate buffer 0.01 M, pH 7.0; citrate buffer 0.01 M, pH 3.0; carbonate buffer pH 9.2. After 2 h hydrogel matrices were transferred to fresh desorption media and the desorption step was repeated till 24 h.

Similarly, in separate experiments, the same amount of loaded hydrogels was placed in 100 mL water solution in the presence of a 12 V external electric voltage for 2 h.

After determination of the released dye in solution by UV-Vis analysis on an Evolution 201 spectrophotometer (ThermoFisher Scientific, Hillsboro, OR, USA), the dye removal (%) was calculated according to the following Equation (7):

(7) $$Recovery\;\left(\%\right)=\frac{D_{t}}{C_{0}-C_{f}}\times 100$$

where *D_t_* is the concentration of dyes in the desorption media, C_0_ and C_f_ the initial and final dyes concentration in the previously performed adsorption process, respectively.

### 3.5. Statistical Analyses

All measurements were done in triplicate and data expressed as means ± SD. Thermodynamics, isothermal and kinetics parameters were calculated by OriginPro 2019 Software (OriginLab Corporation, Northampton, MA, USA).

## 4. Conclusions

Within the present study, experimental evidence that the high adsorption ability of MWCNT and the water affinity of acrylate polymer networks can be effectively combined for the fabrication of hybrid hydrogels suitable for wastewater treatment. More interestingly, the electro-responsivity of MWCNT allowed the modification of sorption capacity and rate upon the application of an external voltage, with the negative polarization of hybrid hydrogels resulting in enhanced or reduced sorption capacity for cationic and anionic (or neutral) molecules, respectively. For MB molecules, the application of the external voltage was found to enhance the $q_{exp}$ from 19.72 mg g^−1^ (0 V) to 33.45 mg g^−1^ (12 V), while a reduction from 28.93 mg g^−1^ (0 V) to 13.06 mg g^−1^ (12 V) and from 36.75 mg g^−1^ (0 V) to 15.85 mg g^−1^ (12 V) was recorded for RD and BR, respectively.

Thermodynamic, isothermal, and kinetics studies showed that (i) the adsorption pro-cesses were possible, spontaneous, and exothermic in all cases, (ii) physical sorbent to sorbate interactions with a predominant surface mechanism are involved; (iii) although predominant sorption processes are involved, the electronic behavior of both hydrogel surface and dye molecules plays a crucial role in determining the sorbent/sorbate interactions.

Regeneration studies showed that the dyes to hydrogel interactions are mainly disrupted in acidic (MB and RD) or alkaline conditions (BR), with the sorption capacity being unchanged after 5 repeated cycles. Furthermore, the reduced affinity of the hybrid hydrogels for RD and BR under 12 V conditions was used as an alternative and effective regeneration method.

Although further experiments are needed to improve the sorbent capacity and investigate the performance in real wastewater sample, overall, our results have shown the potential of the CNT hybrid hydrogel system as a support for wastewater remediation in industrial practice.

## Figures and Tables

**Table 1:** Thermodynamic parameters for MB, RD, and BR adsorption by HG_B_ and HG_NT_.

Dye	HG_B_							HG_NT_						
	R^2^	$∆G_{T_{1}}^{0}$	$∆G_{T_{2}}^{0}$	$∆G_{T_{3}}^{0}$	$∆G_{T_{4}}^{0}$	ΔH^0^	ΔS^0^	R^2^	$∆G_{T_{1}}^{0}$	$∆G_{T_{2}}^{0}$	$∆G_{T_{3}}^{0}$	$∆G_{T_{4}}^{0}$	ΔH^0^	ΔS^0^
MB	0.9143	−23.38	−27.72	−30.28	−33.77	−36.65	217.48	0.9283	−25.31	−28.28	−31.49	−36.05	−37.11	223.97
RD	0.9485	−25.77	−27.56	−29.42	−31.17	−6.35	115.74	0.9514	−26.35	−28.29	−30.63	−32.79	−12.14	138.42
BR	0.9492	−29.59	−32.40	−34.83	−36.57	−12.39	152.01	0.9098	−30.73	−33.94	−36.03	−38.22	−13.18	159.21

ΔG^0^ (kJ mol^−1^); ΔH^0^ (kJ mol^−1^); ΔS^0^ (J mol^−1^ K^−1^); T_1_ = 277.15 K; T_2_ = 293.15 K; T_3_ = 310.15 K; T_4_ = 323.15 K.

**Table 2:** HG_B_ and HG_NT_ sorption capacity (mg g^−^^1^) for MB, RD, and BR dyes at 0, 6, and 12 voltage conditions.

Dye	HG_B_					HG_NT_				
	$q_{exp}^{0}$	$q_{exp}^{6}$	$q_{exp}^{12}$	$∆Q^{6}$	$∆Q^{12}$	$q_{exp}^{0}$	$q_{exp}^{6}$	$q_{exp}^{12}$	$∆Q^{6}$	$∆Q^{12}$
MB	15.94 ± 2.3	17.22 ± 2.2	19.51 ± 1.9	8.03	22.39	19.72 ± 1.9	22.55 ± 1.4	33.45 ± 2.4	14.35	69.62
RD	15.71 ± 1.8	15.67 ± 2.5	15.68 ± 2.4	0.25	0.19	28.93 ± 2.0	25.47 ± 1.7	13.06 ± 1.6	11.96	54.86
BR	23.12 ± 2.6	23.01 ± 2.6	22.96 ± 2.5	0.48	0.69	36.75 ± 2.3	30.74 ± 2.0	15.85 ± 2.0	16.35	56.87

**Table 3:** Constants and variables of isotherm models for adsorption process.

Model/Parameter		MB				RD				BR			
		HG_B_		HG_NT_		HG_B_		HG_NT_		HG_B_		HG_NT_	
		0 V	12 V	0 V	12 V	0 V	12 V	0 V	12 V	0 V	12 V	0 V	12 V
Langmuir	R^2^	0.9984	0.9893	0.9912	0.9688	0.9985	0.9874	0.9621	0.9981	0.9879	0.9650	0.9768	0.9874
	*q_max_*	20.19	25.01	23.01	42.17	18.00	18.51	33.91	16.59	24.18	24.69	42.64	16.87
	*k_L_* (10^−2^)	1.35	1.28	2.46	1.82	2.38	1.99	2.57	1.11	7.27	3.84	3.80	2.75
	*R_L_*	0.16	0.16	0.09	0.12	0.10	0.11	0.09	0.18	0.03	0.06	0.06	0.08
Freundlich	R^2^	0.9283	0.8665	0.8191	0.8004	0.8869	0.8116	0.8387	0.9285	0.9173	0.9354	0.9067	0.9867
	*k_F_*	1.54	1.95	3.46	3.74	2.68	2.60	4.58	1.20	7.13	5.12	7.02	2.99
	*1/n_F_*	0.42	0.41	0.32	0.41	0.32	0.32	0.34	0.41	0.22	0.27	0.32	0.29
Red–Pet	R^2^	0.9974	0.9839	0.9875	0.9477	0.9977	0.9811	0.9432	0.9969	0.9858	0.9636	0.9652	0.9994
	*k_RP_*	0.27	0.32	0.57	0.76	0.43	0.37	0.87	0.18	2.24	1.90	1.62	1.14
	*α_RP_* (10^−2^)	1.34	1.28	2.46	1.72	2.38	1.98	2.57	1.09	12.88	18.87	3.80	21.75
	*g*	0.90	0.75	0.86	0.58	0.06	0.80	0.34	0.88	0.06	0.84	0.22	0.80
	*q_RP_*	20.22	24.98	23.15	44.11	18.07	18.70	33.89	16.47	17.40	10.07	42.60	5.24
Sips	R^2^	0.9994	0.9989	0.9969	0.9939	0.9981	0.9995	0.9747	0.9994	0.9894	0.9691	0.9970	0.9988
	*q_max_*	18.62	21.13	21.00	33.86	17.59	16.07	28.67	15.07	26.36	31.60	36.87	26.79
	*k_S_* (10^−2^)	0.86	0.27	0.93	0.15	2.01	0.25	0.27	0.59	11.86	8.57	0.57	7.16
	*n_S_*	1.15	1.47	1.33	1.85	1.06	1.63	1.78	1.19	0.76	0.62	1.75	0.52
Dub–Rad	R^2^	0.9202	0.9450	0.9369	0.9826	0.9285	0.9810	0.9771	0.9397	0.8759	0.8833	0.9818	0.9026
	*q_max_*	14.16	18.19	18.02	31.88	14.14	14.95	27.02	11.87	20.35	19.68	34.93	13.59
	*β_DR_*	0.35	0.48	0.17	0.25	0.16	0.35	0.17	0.60	0.02	0.05	0.08	0.15
	*E*	1.19	1.02	1.70	1.42	1.79	1.20	1.71	0.91	4.56	3.20	2.46	1.82
Temkin	R^2^	0.9977	0.9839	0.9732	0.9647	0.9890	0.9710	0.9480	0.9967	0.9835	0.9759	0.9528	0.9992
	*A_T_*	0.12	0.11	0.26	0.14	0.26	0.20	0.23	0.10	1.69	0.64	0.37	0.38
	*B*	4.59	0.48	4.81	9.90	3.70	3.96	7.45	3.81	3.93	4.46	9.00	3.24

*q_max_* (mg g^−1^); *k_L_* (L mg^−1^); *k_F_* (mg g^−1^(L mg^−1^)^1/*nF*^); *k_RP_* (L mg^−1^); *α_RP_* (L mg^−1^)^g^; *k_S_* ((L mg^−1^)^ns^); β (mol^2^/kJ^2^); *E* (kJ mol^−1^); *A_T_* (L g^−1^); *B* (kJmol^−1^).

**Table 4:** Parameters of the kinetics models applied to the adsorption processes.

Model/Parameter		MB				RD				BR			
		HG_B_		HG_NT_		HG_B_		HG_NT_		HG_B_		HG_NT_	
		0 V	12 V	0 V	12 V	0 V	12 V	0 V	12 V	0 V	12 V	0 V	12 V
Pseudo-First	R^2^	0.9238	0.9654	0.9500	0.9429	0.9001	0.9050	0.9217	0.9001	0.9528	0.9386	0.9436	0.9226
	*q_e_*	7.43	8.10	10.62	14.94	6.27	5.75	12.37	6.52	14.37	13.11	19.04	9.13
	*k*_1_ (10^−2^)	11.57	11.31	10.87	16.43	6.71	7.21	5.60	4.10	1.38	1.43	1.57	1.55
	$\chi^{2}$	10.63	7.61	11.76	21.72	15.22	14.98	14.26	12.45	0.63	0.58	4.04	8.05
Pseudo-Second	R^2^	0.9699	0.9881	0.9845	0.9740	0.9592	0.9622	0.9739	0.9639	0.9667	0.9561	0.9682	0.9573
	*q_e_*	7.94	8.61	11.32	15.76	6.82	6.24	13.56	7.27	17.17	15.45	22.26	10.60
	*k*_2_ (10^−2^)	1.95	1.82	1.33	1.49	1.30	1.52	0.54	0.73	0.09	0.11	0.09	0.19
	$\chi^{2}$	1.81	0.83	1.53	6.16	2.64	3.10	0.13	0.31	36.38	27.73	24.60	3.53
Avrami	R^2^	0.9711	0.9762	0.9702	0.9614	0.9756	0.9762	0.9889	0.9837	0.9972	0.9732	0.9869	0.9940
	*q_e_*	8.27	11.31	8.38	15.63	7.34	6.55	13.89	7.70	17.83	16.70	24.89	13.47
	*k_A_* (10^−2^)	5.59	8.64	6.90	11.74	2.88	3.37	3.08	1.92	0.77	0.73	0.72	0.49
	*n*	0.47	0.63	0.54	0.54	0.47	0.50	0.54	0.55	0.62	0.59	0.57	0.50
	$\chi^{2}$	0.03	3.02	1.64	7.98	0.13	0.27	0.26	1.04	55.89	65.82	99.32	90.23
Fractional power	R^2^	0.9795	0.9771	0.9915	0.9662	0.9744	0.9689	0.9514	0.9824	0.9599	0.9524	0.9701	0.9812
	*q_e_*	8.29	9.08	11.87	16.36	7.36	6.71	14.51	7.73	15.92	14.53	21.39	10.26
	*k_p_*	3.76	4.26	5.36	8.04	2.17	2.07	3.91	1.67	1.61	1.57	2.46	1.18
	*V*	0.13	0.13	0.14	0.12	0.21	0.20	0.22	0.26	0.39	0.38	0.37	0.37
	$\chi^{2}$	0.01	0.43	0.15	0.91	0.19	0.02	4.51	1.21	9.67	9.02	10.19	0.72
Intraparticle Diffusion	R^2^	0.8008	0.7391	0.7662	0.8085	0.8338	0.8205	0.8336	0.8997	0.9550	0.9466	0.9559	0.9666
	*q_e_*	9.59	10.40	13.61	19.08	8.03	7.37	15.74	8.32	16.55	15.15	22.39	10.72
	*k_i_*	0.41	0.43	0.57	0.82	0.31	0.28	0.63	0.35	0.81	0.74	1.08	0.51
	C	1.80	2.25	2.78	3.53	2.10	1.99	3.80	1.63	1.13	1.19	1.94	0.97
	$\chi^{2}$	16.87	22.18	25.72	28.72	7.64	6.40	26.63	9.71	21.19	20.72	27.24	5.07
Elovich	R^2^	0.9782	0.9660	0.9766	0.9513	0.9927	0.9923	0.9900	0.9956	0.9085	0.9121	0.9555	0.9210
	*α*	4.89	5.31	6.39	21.59	2.73	2.81	3.75	1.24	0.85	0.91	1.35	0.68
	*Β*	0.84	0.78	0.58	0.48	0.96	1.06	0.46	0.79	0.36	0.42	0.26	0.57

*q_e_* (mg g^−^^1^); *k*_1_ (min^−^^1^); *k*_2_ (g mg^−^^1^ min^−^^1^); *k_A_* (min^−^^1^), *k_p_* (mg g^−^^1^ min^−^^ν^); *k_i_* (mg g^−^^1^min^−^^1/2^); *α* (mg g^−^^1^ min^−^^1^); *β* (g mg^−^^1^).

## Data Availability

Not applicable.

## Supplementary Materials

The following are available online, Figure S1: FT-IR of HGNT samples before (black line) and after (red line) the five sorption cycles, Figure S2: Surface appearance of HG_NT_ (a) before and (b) after the five sorption cycles, Figure S3: SEM image of HGNT sample after the five sorption cycles, Figure S4: Raman patterns of HG_NT_ sample after the five sorption cycles Equations (S1)–(S12): Isothermal studies, Equations (S13)–(S19): Kinetics studies, Equations (S20)–(S24): Mean diameter of hydrogels pore, Equations (S25)–(S26): Batch kinetics and equilibrium adsorption studies.
