# Supplementary material for: Carbon Nanotubes Hybrid Hydrogels for Environmental Remediation: Evaluation of Adsorption Efficiency under Electric Field
Source: Molecules. 2021 Nov 19;26(22):7001. doi: 10.3390/molecules26227001 (PMC8625859; doi:10.3390/molecules26227001)
Supplement: Supplementary file 1 [file molecules-26-07001-s001.zip › molecules-1426271-supplementary.pdf]

Supplementary Materials

Carbon Nanotubes hybrid hydrogels for environmental remediation: evaluation of adsorption efficiency under electric field

Giuseppe Cirillo[1,*], Manuela Curcio[1], Lorenzo Francesco Madeo[2], Francesca Iemma[1], Giovanni De Filpo[3], Silke Hampel[2] and Fiore Pasquale Nicoletta[1]

[1] Department of Pharmacy, Health and Nutritional Sciences, University of Calabria, 87036 Rende (CS), Italy
[2] Leibniz Institute of Solid State and Material Research Dresden, 01069 Dresden, Germany
[3] Department of Chemistry and Chemical Technologies, University of Calabria, 87036 Rende (CS), Italy
* Correspondence: giuseppe.cirillo@unical.it; Tel.: +39 0984493208

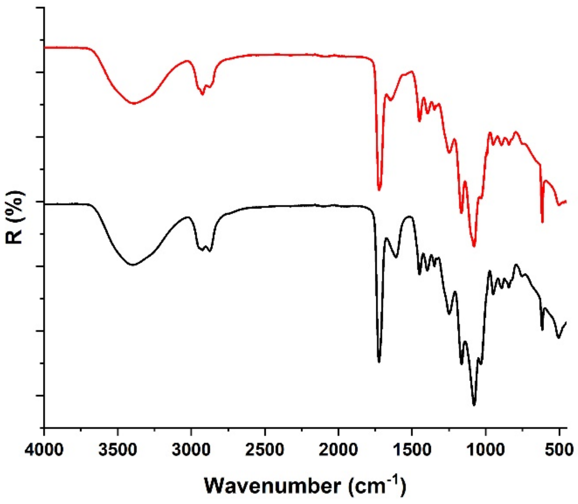

Figure S1. FT-IR of HG$_{NT}$ samples before (black line) and after (red line) the five sorption cycles.

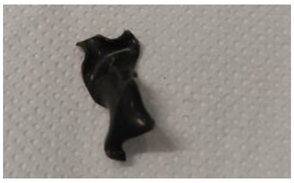

(a)

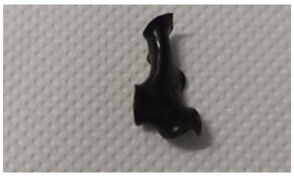

(b)

Figure S2. Surface appearance of HG$_{NT}$ (**a**) before and (**b**) after the five sorption cycles.

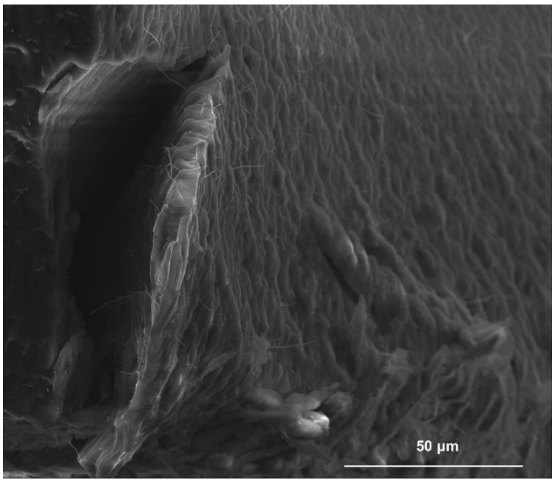

Figure S3. SEM image of HG_{NT} sample after the five sorption cycles.

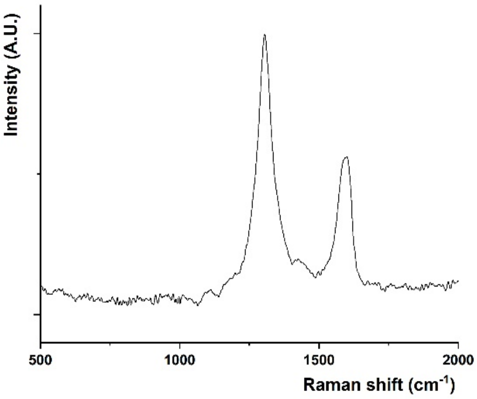

Figure S4. Raman patterns of HG_{NT} sample after the five sorption cycles.

Isothermal studies

Isothermal studies were conducted employing Langmuir (Equation S1), Freundlich (Equation S2), Redlich-Peterson (Red–Pet, Equation S3), Sips (Equation S4), Dubinin–Radushkevich (Dub–Rad, Equation S5), and Temkin (Equation S6) models.

Langmuir $$q_e = \frac{q_{max} k_L C_e}{1 + k_L C_e} \quad \text{(S1)}$$

Freundlich $$q_e = k_F C_e^{1/n_F} \quad \text{(S2)}$$

Red–Pet $$q_e = \frac{k_{RP} C_e}{1 + \alpha_{RP} C_e^g} \quad \text{(S3)}$$

Sips $$q_e = \frac{q_{max} k_S C_e^{n_S}}{1 + k_S C_e^{n_S}} \quad \text{(S4)}$$

Dub–Rad $$q_e = q_{max} e^{(-\beta_{DR} \epsilon)^2} \quad \text{(S5)}$$

Temkin $$q_e = B ln(A_T C_e) \quad \text{(S6)}$$

where q_{max} denotes maximum adsorption capacity of adsorbent (mg g^{-1}), C_e the dye concentration at equilibrium (mg L^{-1}), k_L, k_F, k_s, and A_T the Langmuir, Freundlich, Sips, and Temkin constants, n_F and n_S the Freundlich and the Sips exponents; k_{RP} and α_{RP} the characteristic parameters of the Red–Pet isotherm, β_{DR} and ε the activity and Polanyi coefficients of Dub–Rad isotherm.

The equilibrium parameter R_L of Langmuir model was calculated according to the Equation (S7):

$$R_L = \frac{1}{1+k_L C_0} \quad \text{(S7)}$$

The Specific Surface Area (SSA) was calculated according to the following equation (S8):

$$SSA = \frac{N_A A_D (C_0 - C_e) V}{M_D M_S} \quad \text{(S8)}$$

Where N_A is the Avodadro's number (6.023 10^{23} mol^{-1}), A_D is the covered area per dye molecule, C_0 and C_e the initial and equilibrium dyes concentration, V the volume of dyes solution used, M_D the relative molecular mass of dyes, M_S the mass of samples.

The sorption capacity q_{RP} of Red-Pet model was calculated according to the following equation (S9):

$$q_{RP} = \frac{k_{RP}}{\alpha_{RP}} \quad \text{(S9)}$$

Polanyi coefficients of Dub–Rad isotherm was calculated according to the following Equation (S10):

$$\varepsilon = RTln(1 + \frac{1}{C_e}) \quad \text{(S10)}$$

The apparent energy of adsorption mechanism (E) of Dub–Rad model was calculated according to the following equation (S11):

$$E = (2\beta)^{-1/2} \quad \text{(S11)}$$

The free energy of the sorption process (B) of Temkin model was calculated according to the following equation (S12):

$$B = \frac{RT}{b_T} \quad \text{(S12)}$$

Where b_T is a Temkin constant.

Kinetic studies

Kinetics studies were conducted using six models describing pseudo-first order (Equation S13), pseudo second-order (Equation S14), Avrami (Equation S15), fractional power (Equation S16), intraparticle diffusion (Equation S17), and Elovich (Equation S18) kinetics:

Pseudo-first order	$q_t = q_e(1 - e^{-k_1 t})$	(S13)
Pseudo-second order	$q_t = \frac{k_2 q_e^2 t}{1+k_2 q_e t}$	(S14)
Avrami	$q_t = q_e[1 - e^{[-(k_A t)^n]}]$	(S15)
Fractional power	$q_t = k_p t^v$	(S16)
Intraparticle diffusion	$q_t = k_i t^{1/2} + C$	(S17)
Elovich	$q_t = \frac{1}{\beta} \ln(\alpha\beta t)$	(S18)

with q_e being the adsorption capacity of adsorbent at equilibrium (mg g^{-1}), k_1, k_2, k_A, k_p, and k_i the kinetic constants of pseudo-first order, pseudo-second order, Avrami, fractional power, and intraparticle diffusion models, n and v the Avrami and fractional power exponents, α and β imply the initial absorption and the desorption rates of Elovich model

χ^2 values were calculated according to the following Equation (S19)

$$\chi^2 = \sum_{i=1}^{n} \frac{(q_{exp} - q_e)^2}{q_e} \times 100 \quad \text{(S19)}$$

Mean diameter of hydrogels pore

The mean diameters of hydrogels pore (ξ) were determined by applying the empirical model proposed in the literature [37] according to the following Equation (S20):

$$\xi = 0.071\phi^{-1/3}(\bar{M}_c)^{1/2} \quad \text{(S20)}$$

Here, $\bar{M}_c$, the molecular weight of the polymer chain between two neighbouring crosslinking points, is calculated according to Equation (S21):

$$\bar{M}_c = -d_p v_{m,1} \phi^{1/3} [\ln(1-\phi) + \phi + \chi\phi^2]^{-1} \tag{S21}$$

$v_{m,1}$ represents the molar volume of the swelling media, while χ and ϕ are the Flory–Huggins interaction parameter and the polymer volume fraction in the swollen state, respectively.
ϕ is calculated by following Equation (S22):

$$\phi = \left[\left(\frac{d_p}{d_s}\right)\left(\frac{W_s - W_d}{W_d}\right) + 1\right]^{-1} \tag{S22}$$

d_p and d_s are the densities of polymer and solvent; W_d and W_s the weight of polymer before and after 3 h swelling, respectively.
χ was calculated experimentally from the temperature coefficient of volume fraction ($d\phi/dT$) according to Equation (S23):

$$\chi = [\phi(1-\phi)^{-1} + N\ln(1-\phi) + N\phi]\left[2\phi - \phi^2 N - \phi^2 T^{-1}\left(\frac{d\phi}{dT}\right)^{-1}\right]^{-1} \tag{S23}$$

Here, ($d\phi/dT$) is the slope obtained by plotting the volume fraction data versus temperature (in K), while N is calculated according to Equation (S24):

$$N = \left(\frac{\phi^{2/3}}{3} - \frac{2}{3}\right)\left(\phi^{1/3} - \frac{2}{3}\phi\right)^{-1} \tag{S24}$$

Batch kinetics and equilibrium adsorption studies

The adsorption capacity at time t (q_t) and at equilibrium (q_e) were expressed using the following Equation (S25) and Equation (S26):

$$q_t = \frac{C_0 - C_t}{m} \times V \tag{S25}$$

$$q_e = \frac{C_0 - C_e}{m} \times V \tag{S26}$$
